# Preponderance of microbial isolates among heart transplantation recipients requiring renal replacement therapy: a propensity score-adjusted analysis

**DOI:** 10.3325/cmj.2018.59.224

**Published:** 2018-10

**Authors:** Hrvoje Gašparović, Lucija Svetina, Filip Lončarić, Jana Ljubas, Maja Čikeš, Bojan Biočina, Davor Miličić

**Affiliations:** 1 Department of Cardiac Surgery, University Hospital Center Zagreb, Zagreb, Croatia; 2 Department of Cardiology, University Hospital Center Zagreb, Zagreb, Croatia; * HG and LS contributed equally.

## Abstract

**Aim:**

To assess the association between renal replacement therapy (RRT) and post-transplant infection incidence.

**Methods:**

This single-center retrospective cohort study included 158 patients who underwent heart transplantation (HTx) in our center from 2008 to 2016, survived beyond the first post-procedural day, and had available microbial data. The patients were dichotomized according to the need for periprocedural RRT. Twenty-seven patients in RRT group had lower preoperative creatinine clearance, greater body mass index, and higher likelihood of having diabetes. Propensity score adjustment was used to account for multiple covariates. The primary outcome measure was the presence of bacteremia in patients with and without the need for RRT. The secondary outcome measures were the presence of microbial isolates from any culture and clinical outcome data.

**Results:**

Unadjusted analysis showed that the RRT group had higher incidence of any positive microbial isolate (93% vs 73%; odds ratio [OR] 4.77, 95% confidence interval [CI] 1.01-30.53; *P* = 0.026) and an increased susceptibility to bacteremia (50% vs 22%; OR 3.50, 95% CI 1.28-9.67; *P* = 0.012). Propensity score-adjusted analysis corroborated the between-group difference in positive blood cultures (OR 3.97, 95% CI 1.28-12.32; *P* = 0.017). There was no difference in the incidence of total microbial isolates between the groups (OR 4.55, 95% CI 0.90-23.05; *P* = 0.067).

**Conclusions:**

Patients requiring RRT after HTx had an increased susceptibility to infections via various portals of entry, predominantly due to an increase in blood-borne infections. Understanding the underlying conditions leading to infection-related morbidity is important for infection control and prevention.

Heart failure (HF) definition is a moving target, as new insights into the pathophysiology become available and novel markers allow for identification of subclinical HF (1). As the projected number of patients with HF increases, so does the number of its treatment options. Heart transplantation (HTx) has, however, remained the benchmark with which all other therapies are compared (2,3).

Among the most important predictors of adverse outcomes after HTx are systemic infections (4). The infection risk in these patients is promoted by factors such as immunosuppression, prolonged use of indwelling cannulas, hemodynamic compromise antedating the HTx, and surgical trauma. Additionally, infections are the second most common cause of death in patients requiring renal replacement therapy (RRT). In these patients, the presence of positive microbial isolates carries a significantly worse prognosis, irrespective of solid organ transplantation. The risk stems from the use of intravascular instrumentation, blood product consumption, and disorders of innate and adaptive immunity (5).

The unique features of HTx recipients in combination with RRT lead to compounding of predisposing conditions for infections, with bloodstream infections (BSI) being clearly associated with mortality (4,6,7). Twenty-six percent of HTx recipients will develop renal dysfunction within the first year (3). Patients requiring RRT therapy after HTx have mortality rates exceeding 50% (8). This is in notable contrast to the mortality rate in patients who do not develop kidney injury (8). Renal insufficiency frequently becomes manifest later in the postoperative course as a consequence of prolonged calcineurin-inhibitors use. Sixteen percent of patients develop renal dysfunction within five years after transplantation and 30% within 10 years after transplantation (9).

Understanding the origins and predisposing conditions is critical for outcome improvement in patients with post-transplantation renal dysfunction. The severity of the underlying disease leading to end-organ failure correlates with the risk of postoperative morbidity and mortality. Similarly, chronic malnutrition and advanced patient age predispose to transplantation-associated infections (10).

Most studies on renal dysfunction in cardiac transplant recipients focus on long-term renal outcomes, with only a few focusing on the early postoperative period (11,12). The impact of renal dysfunction on the infection incidence in the early postoperative period remains largely unknown. Our aim was to evaluate the association between RRT and positive blood cultures in HTx recipients during the first postoperative month. We hypothesized that RRT was associated with bacteremia and decreased survival.

## METHODS

### Study participants

**Figure 1:**
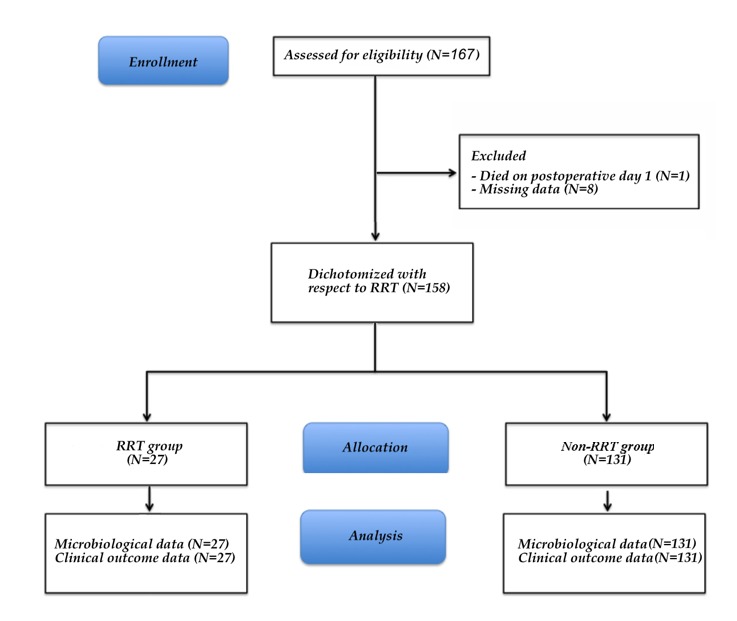
Study flowchart. *RRT – renal replacement therapy.

This retrospective cohort study was conducted at the University Hospital Center Zagreb in Zagreb, Croatia. One-hundred and sixty-seven patients underwent HTx in our academic center from January 2008 to December 2016. The procedures were performed by 6 senior transplant surgeons. The inclusion criterion was heart transplantation performed during the study period. Exclusion criteria were non-transplant cardiac surgical procedures, death within the first post-procedural day, and missing microbial data (n = 9). The remaining 158 patients were dichotomized according to the need for periprocedural RRT (Figure 1). Demographic, clinical, and laboratory data were obtained from a comprehensive database of HTx patients treated in our institution. While RRTs encompass multiple options, they all share the goal of fluid removal and solute clearance and are, therefore, presented collectively in our study. Patients who did not require RRT postoperatively (non-RRT group) had significantly different preoperative profiles from those who required RRT (RRT group) (Table 1).

**Table 1:** Preoperative variables in heart transplant recipients dichotomized with respect to renal replacement therapy (RRT)

	No. (%) of patients		
Patient characteristics	RRT group (n = 27)	non-RRT group (n = 131)	P*
Age (years; median and range)	57 (14-66)	54 (5-70)	0.425
Men	24 (89.0)	96 (73.0)	0.117
Pulmonary vascular resistance (dyn · s · cm^−5^; mean ± standard deviation)	198 ± 105	209 ± 99	0.371
Body mass index (kg/m^2^; mean ± standard deviation)	27 ± 3	25 ± 5	0.014
Ischemic cardiomyopathy	13 (48.0)	42 (32.0)	0.124
Dilated cardiomyopathy	13 (48.0)	73 (56.0)	0.579
Diabetes mellitus	11 (41.0)	26 (20.0)	0.026
Hyperlipidemia	7 (26.0)	41 (31.0)	0.701
Preoperative hypertension	9 (33.0)	43 (33.0)	0.998
Chronic obstructive pulmonary disease	1 (4.0)	6 (5.0)	1.0
Atrial fibrillation	10 (37.0)	44 (34.0)	0.789
History of smoking	5 (19.0)	19 (15.0)	0.565
Preoperative beta-blocker	13 (48.0)	64 (49.0)	0.999
Preoperative amiodaron	9 (33.0)	41 (31.0)	0.854
Preoperative aspirin	7 (26.0)	23 (18.0)	0.296
Creatinine clearance (mL/min; mean ± standard deviation)^†^	53 ± 21	62 ± 21	0.044
Moderate or greater kidney dysfunction	18 (67.0)	61 (47.0)	0.067
Reoperation	10 (37.0)	31 (24.0)	0.156
Preoperative mechanical circulatory support	4 (15.0)	17 (13.0)	0.760

*Mann-Whitney U test was used for continuous variables and the Fisher exact test for categorical variables.

†Defined as creatinine clearance <60 mL/min.

To account for these discrepancies and elucidate the independent associations between the need for RRT and infection, we performed a propensity score-adjusted analysis. The covariates used in the adjusted analysis included recipient age and sex, organ ischemic time, body mass index, pulmonary vascular resistance, creatinine clearance, duration of cardiopulmonary bypass, diabetes, and preoperative and postoperative mechanical circulatory assistance. The primary outcome measure was the presence of positive blood cultures within the first postoperative month. The secondary outcome measures were the presence of any microbial isolates within the first postoperative month and individual components of this composite outcome. Furthermore, overall clinical outcomes adjudicated at 3 months postoperatively are presented, as is mortality among patients with positive blood cultures dichotomized with respect to RRT.

The attending microbiologists evaluated all microbiological data. Cultures considered to be contaminates were excluded from the analysis.

### Heart transplantation

The immunosuppression regime included oral prednisone, calcineurin inhibitors (tacrolimus or cyclosporine A [CyA]) and mycophenolate mofetil) (13). All patients received induction therapy with antithymocyte globulin. All transplant recipients routinely received antimicrobial prophylaxis with vancomycin and meropenem until chest tube removal. For oral candidiasis, patients received miconazole gel; for *Pneumocystis carinii* prophylaxis, trimethoprim sulfamethoxazole; and for cytomegalovirus prophylaxis, ganciclovir followed by valganciclovir (which has improved oral bioavailability). The bicaval technique for HTx was used in 137 (87%) patients and biatrial HTx in 21 (13%) patients. All patients received intra-arterial catheters for continuous systemic blood pressure monitoring as part of comprehensive intraoperative monitoring. Furthermore, three-luminal central venous catheters were inserted via the internal jugular vein and a Swan-Ganz catheter, used for continuous pulmonary arterial pressure and intermittent pulmonary capillary wedge pressure monitoring, was placed into the pulmonary artery in every patient. Cardiac performance was evaluated via thermodilution measurements. All patients underwent intraoperative transesophageal echocardiography.

### Microbial culture sampling

All patients were continuously monitored for the presence of clinically relevant infections. Patients in whom an infection was clinically suspected or established were pancultured (paired blood cultures, bronchial aspirates, wound discharge cultures [if present], and urine cultures) and underwent appropriate imaging procedures, including chest x-rays, computed tomographic imaging, or echocardiography. Bronchial aspirates and urine cultures were taken in all patients upon arrival to the intensive care unit. Some samples were subsequently disregarded if they were considered to be contaminated. Urine cultures are not presented because the RRT group had diminished or non-existent urine output, which makes the between-group comparison impossible. One-hundred and sixteen patients had at least one blood culture (90 [69%] in the non-RRT group and 26 [96%] in the RRT group). The analysis also included 133 bronchial aspiration cultures (106 [81%] in the non-RRT group and 27 [100%] in the RRT group) and 54 wound cultures (43 [33%] in the non-RRT group and 11 [41%] in the RRT group).

### Statistical analysis

Continuous data are presented as mean ± standard deviation or medians with ranges. Normality of distribution was assessed using the Shapiro-Wilk test. The Mann-Whitney U test was used for continuous data testing. Categorical variables and endpoints are presented as absolute numbers with percentages and were compared across groups using 2 × 2 contingency tables. Measures of association were derived from the Fisher exact test. A two-tailed *P* < 0.05 was considered significant. Statistical analysis was performed using the Statistica v. 13.0 software package (Dell, Round Rock, TX, USA, licensed to the University of Zagreb School of Medicine).

## RESULTS

### Study population

Of 158 HTx recipients, 27 (17%) required RRT. Preoperative comorbid factors favoring postoperative RRT requirement were higher body mass index, diabetes mellitus, and lower preoperative renal reserve (Table 1). Recipient age and sex, as well as duration of organ ischemia, cardiopulmonary bypass, and total operative times were comparable between the groups. Clinical outcome data are presented in Table 2. Sex mismatch between the recipients and the donor hearts was present in 49 (31%) of 158 HTx recipients. It did not influence any of the outcomes. There was no difference in the incidence of positive microbial isolates between patients receiving sex-mismatched organs and patients receiving sex-matched organs (35/49 [71%] vs 85/109 [78%], respectively; *P* = 0.403). Similarly, the incidences of RRT requirement (7/49 [14%] vs 20/109 [18%], respectively; *P* = 0.689) and 3-month mortality (9/49 [18%] vs 13/109 [12%], respectively, *P* = 0.336) were unaffected by sex mismatch.

**Table 2:** Perioperative variables and clinical outcome data in heart transplant recipients dichotomized with respect to renal replacement therapy (RRT)

	No. (%) of patients		
Perioperative variables	RRT group (n = 27)	non-RRT group (n = 131)	P*
Organ ischemia (min, mean ± standard deviation)	179 ± 65	183 ± 65	0.801
Cardiopulmonary bypass (min, mean ± standard deviation)	184 ± 78	164 ± 71	0.247
Duration of surgery (min, median and range)	445 (270-780)	420 (190-900)	0.261
Recipient-donor sex mismatch	7 (26.0)	42 (32.0)	0.590
Mechanical ventilation (h, median and range)^†^	252 (24-680)	24 (3-984)	<0.001
Postoperative mechanical circulatory support	11 (41.0)	8 (6.0)	<0.001
Cyclosporine concentration (ng/mL; median and range)	144 (43-223)	216 (60-395)	<0.001
Tacrolimus concentration (ng/mL; median and range)	13.6 (9.2-22.5)	12.7 (4.1-27.1)	0.449
**Clinical outcome**			
Stroke	3 (11.0)	5 (4.0)	0.138
Resternotomy	13 (48.0)	13 (10.0)	<0.001
3-month mortality	17 (63.0)	5 (4.0)	<0.001

*Mann-Whitney U test was used for continuous variables and the Fisher exact test for categorical variables.

### Immunosuppression

Our institutional protocol mandates concentration-controlled mycophenolate mofetil immunosuppression in a long-term follow-up but not in the early postoperative management. Calcineurin inhibitor immunosuppression is, however, dose-controlled as soon as it is started. We did not observe a significant difference in the CyA values between patients who had positive microbial isolates and those who did not (201 [43-349] ng/mL vs 215 [150-395] ng/mL, respectively; *P* = 0.072). Similarly, we did not observe a difference in tacrolimus values between patients who had positive microbial isolates and those who did not (13.8 [4.9-27.1] ng/mL vs 11.5 [10.3-13.3] ng/mL, respectively; *P* = 0.255). Values most proximal to the end of the studied postoperative course are presented. We did, however, observe significantly higher CyA concentrations in patients who did not require RRT (Table 2). Corticosteroid levels were not monitored.

### Microbial pathogen isolates

The unadjusted univariate analysis showed that the patients in RRT group were significantly more likely to have positive microbial culture isolates from any source and from blood cultures in comparison with patients in non-RRT group (Table 3). Propensity score-adjusted analysis corroborated the significant between-group difference with respect to positive blood cultures (OR 3.97, 95% CI 1.28-12.32; *P* = 0.017), but showed no significant difference between the groups with respect to total microbial isolates (OR 4.55, 95% CI 0.90-23.05; *P* = 0.067). No significant differences in the proportions of positive microbial cultures harvested from the bronchial aspirates and wounds were noted between the groups.

**Table 3:** Primary and secondary study outcomes in univariate analysis of postoperative microbial isolates in heart transplant recipients dichotomized with respect to renal replacement therapy (RRT)

	No. (%) of patients			
Primary outcome	RRT group	non-RRT group	Odds ratio (95% confidence interval)	P*
No. of patients with positive blood culture^†^	13/26 (50.0)	20/90 (22.0)	3.50 (1.28-9.67)	0.012
**Secondary outcomes**				
No. of patients with positive microbial isolates^‡^	25/27 (93.0)	95/131 (73.0)	4.77 (1.01-30.53)	0.026
No. of patients with positive bronchial aspirate^§^	23/27 (85.0)	73/106 (69.0)	2.60 (0.77-9.68)	0.147
No. of patients with positive wound culture^‖^	8/11 (73.0)	18/43 (42.0)	3.70 (0.74-20.78)	0.095

*Fisher exact test.

There was no difference in the proportion of microbial isolates across the Gram stain spectrum between the groups (Table 4). Similarly, the use of oxygen for bacterial metabolism did not differ between the groups. The majority of the isolates belonged to facultative aerobes, especially in the non-RRT group. None of the comparisons in microbial distribution showed a pattern that could be related to RRT (Table 4).

**Table 4:** Distribution of Gram positive and Gram negative blood culture isolates in heart transplant recipients dichotomized with respect to renal replacement therapy (RRT)

	No. of blood culture isolates		
Microbial isolates	RRT group*	non-RRT group^†^	*P^‡^*
Gram positive	9 (45)	16 (57)	0.559
Gram negative	11 (55)	12 (43)	0.559
Obligate aerobes	4 (20)	2 (7)	0.218
Anaerobes	3 (15)	4 (14)	>0.1
Facultative anaerobes	13 (65)	22 (79)	0.339

*20 blood culture isolates from 13 patients.

†28 blood culture isolates from 20 patients.

‡Fisher exact test.

### Clinical outcomes

The incidence of postoperative mechanical circulatory assistance in the RRT group was 41%, compared with only 6% in the non-RRT group (OR 10.57, 95% CI 3.31-34.54; *P* < 0.001). Patients in the RRT group were also significantly more likely to require prolonged inotropic or vasoactive support (Table 5).

**Table 5:** Prolonged inotropic support requirement defined as the need for inotropes for 7 or more days in heart transplant recipients dichotomized with respect to renal replacement therapy (RRT)

	No. (%) of patients		
Inotrope/ vasoactive medication	RRT group (n = 27)	non-RRT group (n = 131)	P*
Dobutamine	7 (26)	8 (6)	0.005
Epinephrine	15 (56)	18 (14)	<0.001
Isoproterenol	17 (63)	68 (52)	0.349
Norepinephrine	8 (30)	16 (12)	0.04
Levosimendan	3 (11)	8 (6)	0.402
Milrinone	3 (11)	25 (19)	0.415

*Fisher exact test.

The increase in mortality was robust and highly significant (17/27 [63%] vs 5/131 [4%], OR 42.84, 95% CI 11.58-170.87; *P* < 0.001). The mortality of patients with positive blood cultures was significantly higher in RRT group than in non-RRT group (8/13 [62%] vs 1/20 [5%], OR 30.40, 95% CI 2.58-828.98; *P* = 0.001). Clinical outcome data are summarized in Table 2.

## DISCUSSION

We showed that RRT following HTx was associated with an increased susceptibility to infections via various portals of entry, predominantly due to an increase in blood-borne infections.

Understanding the underlying conditions leading to infection-related morbidity is paramount to infection control and prevention. Infectious complications remain among the leading causes of inferior clinical outcomes in patients with solid organ transplantation (14). Bacteremia-associated annual mortality is 100-300 times greater in RRT patients compared with the general population irrespective of solid organ transplantation (14). Infections after HTx account for 30% of all deaths within the first postoperative year of transplantation (15). This higher infection rate can be attributed to both higher immunosuppression levels and the temporal proximity to surgical disruption of anatomical barriers (15). Certain predisposing risk factors are modifiable, while others are not subject to change. Reduction of the immunosuppression dosing may reduce infections and mortality incidence, but at the expense of increased rejection risk (11). Surgical predisposing factors involved are disruption of both allograft vascular supply and functional integrity. There is also a clear correlation between the surgery duration and infection incidence (16).

We specifically examined the impact of RRT on the incidence of new-onset positive microbial isolates from a variety of sampling regions. The observed RRT incidence of 17%, which resulted in a 63% mortality rate, is in line with previous reports (9). Clearly, kidney dysfunction in these patients may only be a measurable metric of multi-organ failure and not a problem in and of itself. Isolated acute renal failure is more likely to occur in patients with reduced preoperative renal functional reserve (17). The interdependent relationship between the heart and kidney in HTx candidates is invariably compromised. The ensuing cardiorenal syndrome results in chronic kidney disease, which was common in our patient population. An additional attribute of the HTx population requiring RRT was that the optimization of their hemodynamics required prolonged inotropic support. This underscores their inherent postoperative hemodynamic compromise, which, coupled with the prolonged use of intravascular instrumentation required for RRT and drug delivery, likely enhanced the susceptibility to infections. We found higher CyA levels in patients who did not require RRT, likely due to the fact that CyA doses were purposefully reduced in patients with signs of acute kidney injury in order to reduce the nephrotoxic impact of CyA.

The focus of our study was on bacterial microbial isolates since these infections predominate in the immediate perioperative period, as opposed to viral, opportunistic, and fungal infections, which predominate later on.

In a Spanish study from the RESITRA cohort, the BSI incidence in HTx recipients was 11% (4). Rodriguez et al (16) reported 60 BSI episodes in 15.8% of patients in the postoperative period. It is important to note that over 50% of all bacterial infections occur within the first postoperative month (8). We performed microbiological surveillance sampling systematically, irrespective of clinical indications, as a part of our standard institutional protocol. Therefore, not all positive microbial culture isolates resulted in clinically relevant infections.

Bacteremia is most commonly associated with vascular access and especially with central venous catheters use, with 32% of hospitalizations for vascular access infection occurring in patients with a central venous catheter in place (5). Most patients develop at least one serious periprocedural complication (serious infection, cardiac morbidity, or neurological morbidity) before requiring RRT. In contrast, only 17% of patients develop severe renal injury requiring RRT without adjoining non-renal comorbidities, which is consistent with our results. Only one previous study identified RRT to be an independent risk factor for bacteremia in HTx patients (16). The authors concluded that immune alterations produced by renal failure and subsequent RRT were responsible for the increased risk of adverse outcomes. Independent risk factors for BSI were prolonged intensive care unit stay and previous cytomegalovirus infection (16). In liver and kidney transplant patients, bacteremia is a predictor of higher mortality up to two months after transplantation (16). Regional bacterial epidemiology should be closely followed and appreciated in order to design the appropriate antibiotic prophylaxis for solid organ transplantation

A limitation of our study is bias in data selection and analysis stemming from the study’s retrospective design. Another limitation is incompleteness of data input, leading to possible underappreciation of some confounding variables. Patients experiencing a complicated postoperative course will have a proportionally greater need for a variety of diagnostic and therapeutic procedures (analysis of microbiological samples included). This could enhance the probability of diagnosing an incipient microbial isolate in the RRT group, which would have otherwise not been identified had the postoperative course been uneventful.

In conclusion, we found that patients requiring RRT had markedly more complicated postoperative courses. We also documented a significant relationship between RRT and an increased infection risk. Our data add to the literature on the subject of early post-transplantation infection burden. Measures designed to reduce the bacteremia incidence should be rigorously implemented in heart transplant recipients, and especially in patients with increased susceptibility to renal failure.

## 

†26 patients in the RRT group and 90 patients in the non-RRT group had at least one blood sample cultured.

‡27 patients in the RRT group and 131 patients in the non-RRT group had at least one microbial sample cultured.

§27 patients in the RRT group and 106 patients in the non-RRT group had at least one bronchial aspirate cultured.

‖11 patients in the RRT group and 43 patients in the non-RRT group had at least one wound culture taken.
